# Correction: Neural excitability and sensory input determine intensity perception with opposing directions in initial cortical responses

**DOI:** 10.7554/eLife.76898

**Published:** 2022-01-19

**Authors:** Tilman Stephani, Alice Hodapp, Mina Jamshidi Idaji, Arno Villringer, Vadim Nikulin

**Keywords:** Human

Stephani T, Hodapp A, Jamshidi Idaji M, Villringer A, Nikulin VV. 2021. Neural excitability and sensory input determine intensity perception with opposing directions in initial cortical responses. *eLife* **10**:e67838. doi: 10.7554/eLife.67838

Published 5, October 2021

After publication, we noticed that an oversight had occurred during our internal review process that affected the description of the presented somatosensory stimuli. Electrical stimuli were presented – as intended – with a duration of 200 µs, while the original text stated a stimulus duration of 20 µs.

This error neither affects the results nor the conclusions of the article but should be corrected in order to ensure the exact replicability of the experimental design.

We regret this inaccuracy and have corrected the manuscript as follows:

Original text (page 14, lines 2–3 of the section "Stimuli"):

"The electrical stimuli were designed as squared pulses of a 20 µs duration [...]."

Corrected text:

"The electrical stimuli were designed as squared pulses of a 200 µs duration [...]."

The article has been corrected accordingly.

