# Supplementary material for: Neural excitability and sensory input determine intensity perception with opposing directions in initial cortical responses
Source: eLife. 2021 Oct 5;10:e67838. doi: 10.7554/eLife.67838 (PMC8492057; doi:10.7554/eLife.67838)
Supplement: Figure 2—source data 1. — Trial overlap between extreme bins of N20 and pre-stimulus alpha amplitudes used for the SDT analyses. [file elife-67838-fig2-data1.pdf]

	N20: most negative 20%	N20: least negative 20%
Alpha: lowest 20%	19.45%	21.33%
Alpha: highest 20%	21.16%	18.83%
